# *Teucrium polium* and kidney

**DOI:** 10.12861/jrip.2013.02

**Published:** 2013-03-01

**Authors:** Mahmoud Rafieian-Kopaei, Azar Baradaran

**Affiliations:** ^1^Medical Plants Research Center, Shahrekord University of Medical Sciences, Shahrekord, Iran; ^2^Department of Clinical Pathology, Isfahan University of Medical Sciences, Isfahan, Iran

**Keywords:** *Teucrium polium*, Diabetes, Kidney injury

**Implication for health policy/practice/research/medical education:**

Decision about the use of a drug on prevention or amelioration of diseases requires knowledge about the side effects on different organs, specially kidney or liver.

An article recently published entitled “Preventive effect of *Teucrium polium* on learning and memory deficits in diabetic rats” (1). They aimed to test passive avoidance learning and memory in control and streptozocin-induced diabetic rats, while they were under chronic treatment with an aqueous extract of *Teucrium polium*. Impairment in acquisition of passive avoidance learning and retrieval of memory was seen by inducing diabetes. *Teucrium polium* treatment (200 and 400 mg/kg) improved learning and memory in control rats and reversed learning and memory deficits in diabetic rats. They concluded that *Teucrium polium* prevented the deleterious effects of diabetes on passive avoidance learning and memory. They suggested that, antioxidant, anticholinesterase and hypoglycemic effects of Teucrium might be involved in the obtained effects. They also concluded that, *Teucrium polium* appears to be a promising candidate for memory improvement in diabetes (1). At this paper, we would like to point out a few comments. In the use of any drug, it is very important to consider its adverse reactions. An adverse drug reaction (ADR) is a response to a medicine which is noxious and unintended, and which occurs at doses normally consumed in man (2). About 6% of hospital admissions (3) and 2.5% of emergency department visits for poisonings (4) may be due to adverse drug reactions. Adverse drug reactions also happen among ambulatory outpatients (5) and among inpatients (6). A minority of drug toxicity is recognized by health care providers (7). Medicinal plants are considered to be safe in comparison to synthetic drugs, however, it does not mean that their possible toxicities need not to be considered.

To test the possible renal toxicity of hydroalcoholic extract of *Teucrium polium*, we recently studied 100 male Wistar rats (8). Rats were divided into 10 groups of ten each. Five groups were injected intraperitoneally (ip), 50, 100, 150, 200 mg/kg extracts and normal saline for 28 days and killed to examine the probable renal injury. Five other groups were injected the same drug regimen, but they were killed 28 days after discontinuation of *Teucrium polium* injections to find out possible renal complications or regeneration during recovery. Following 28 days of *Teucrium polium* receiving (phase I), renal injury was not increased in comparison with control group. However, following 28 days of drug cessation, kidney injury including degeneration, destruction and vacuolization (9), appeared in comparison to control group. In this study, we concluded that *Teucrium polium* may be associated with kidney tubular injury and this herbal medicine should be used with caution (8,9). It is well known that herbal remedies have an important role in the recovery of some disease (10,11), however, some of the medicinal plant can be a common source of renal injury. *Teucrium polium* has been widely used to control of blood sugar in diabetes, too (1-4). Hence according to our results, concerning its renal tubular cell toxicity, it needs much attention to re-evaluate its use. Indeed decision about the use of a drug on prevention or amelioration of diseases requires knowledge about the side effects on different organs, specially kidney or liver (10,11). In this regard, to better understand the hepatic and renal effects of *T. polium*, more experimental or clinical studies are suggested.

## Authors’ contributions

AB wrote the manuscript. MRK contributed to the final preparation of the manuscript.

## Conflict of interests

None to declare.

## Ethical considerations

Ethical issues (including plagiarism, data fabrication, double publication) have been completely observed by the author.

## Funding/Support

No financial support by any institution.
